# Effects of Feeding Strategies on Gut Microbial Communities in Donkeys: A Comprehensive Narrative Review

**DOI:** 10.3390/vetsci13010007

**Published:** 2025-12-20

**Authors:** Lin Wei, Jinjin Wei, Xiaotong Liu, Wenting Chen, Changfa Wang, Muhammad Zahoor Khan, Zhenwei Zhang

**Affiliations:** College of Agriculture and Biology, Liaocheng University, Liaocheng 252000, China

**Keywords:** donkey nutrition, gastrointestinal microbiota, hindgut fermentation, roughage digestibility, nutritional interventions

## Abstract

Donkeys (*Equus asinus*) remain nutritionally understudied despite their critical roles in agriculture across developing regions, with current feeding practices inappropriately extrapolating horse standards without accounting for species-specific digestive physiology. No comprehensive synthesis has integrated how dietary modifications systematically alter gut microbial communities to drive measurable health outcomes in donkeys, preventing development of evidence-based feeding strategies. This review critically synthesizes current evidence on donkey nutritional requirements and gut microbiota dynamics to establish mechanistic frameworks for optimizing health and productivity. Donkeys exhibit remarkable adaptations including 30% superior fiber digestibility versus horses and specialized hindgut fermentation where microbiota provide 60–70% of metabolic energy. Targeted nutritional interventions—protein supplementation (12.52%), methionine supplementation (5–15 g/day), and optimized energy levels (10.49 MJ/kg)—have shown preliminary evidence of modulating beneficial microbial populations (*Prevotella*, *Ruminococcus*, *Akkermansia*, *Bacteroides*), with short-term studies (typically 30–60 days, *n* < 10 animals) indicating potential reducing oxidative stress (20–40%), decreasing inflammatory cytokines (30–50%), and improving growth performance (15–25%). However, these findings require validation through larger-scale, longer-term studies to establish sustainable effects and broader applicability. Non-conventional feeds including reed silage, bamboo leaves, and garlic byproducts offer sustainable alternatives. This narrative review uniquely establishes mechanistic pathways linking dietary modifications to microbiota changes and downstream physiological effects, providing the first comprehensive framework integrating digestive physiology, microbiota ecology, and nutritional interventions specifically for donkeys to support evidence-based, sustainable feeding strategies.

## Simple Summary

This narrative review examines donkey nutrition through the lens of digestive physiology and gut microbiota interactions. Donkeys demonstrate remarkable evolutionary adaptations to harsh environments, including exceptional fiber digestibility and efficient hindgut fermentation systems where microorganisms provide 60–70% of metabolic energy. We have synthesized evidence on diverse feed resources—from traditional roughages like corn stalks and wheat straw to innovative alternatives such as reed silage, bamboo leaves, and garlic byproducts utilized in equine nutrition. Critically, the review highlights how targeted dietary interventions (protein, methionine, energy optimization) show potential to modulate beneficial microbial populations, with preliminary evidence suggesting reductions in oxidative stress and inflammation in small-scale studies, though long-term effects on growth and immunity in donkeys require further investigation with larger sample sizes and extended study durations. In addition, significant knowledge gaps remain regarding species-specific nutritional standards, necessitating further research to develop evidence-based feeding strategies for sustainable donkey production systems.

## 1. Introduction

Donkeys (*Equus asinus Linnaeus*, *1758*) represent one of humanity’s oldest domesticated animals, with their origins tracing back to the African wild ass approximately 7000 years ago [1,2]. Despite their historical significance and continued importance in agriculture, transportation, and milk production across developing regions, donkey nutrition remains significantly understudied compared to other livestock species [3,4,5]. This knowledge gap is particularly concerning given the growing recognition of donkeys’ economic and social value [6], alongside increasing interest in donkey milk for human consumption and the species’ potential role in sustainable agriculture systems [7,8,9,10,11].

As members of the Equidae family, donkeys are non-ruminant, monogastric herbivores that have evolved sophisticated digestive strategies to extract nutrients from fibrous, low-quality forages [12].Their digestive physiology differs fundamentally from both ruminants and horses, featuring proportionally larger gastrointestinal tracts relative to body size and specialized hindgut fermentation systems centered in the cecum and colon [13,14]. These anatomical adaptations enable donkeys to achieve remarkable digestive efficiency, particularly when processing poor-quality roughages, where they demonstrate up to 30% higher fiber digestibility than horses of comparable size [15,16].

The gastrointestinal microbiota plays a pivotal role in donkey nutrition, facilitating the breakdown of complex carbohydrates into volatile fatty acids that provide 60–70% of the animal’s metabolic energy requirements [17,18,19]. Recent advances in molecular techniques have begun to elucidate the complexity of donkey gut microbial communities, revealing distinct populations across different gastrointestinal segments and significant variation based on geographic location, diet composition, and management practices [20,21,22,23].Understanding these microbiota-nutrition interactions has become increasingly critical as researchers recognize their influence on nutrient utilization, immune function, oxidative stress management, growth performance, and overall animal health [24,25].

Traditional donkey feeding practices have relied heavily on locally available roughages and agricultural byproducts, often without systematic consideration of nutritional adequacy or optimization. While donkeys’ reputation as “easy keepers” reflects their efficiency in utilizing low-quality feeds, this perception has sometimes led to suboptimal nutrition that may compromise welfare and productivity [26].Contemporary approaches to donkey nutrition must balance the species’ evolutionary adaptations with modern understanding of nutritional requirements, feed processing technologies, and the economic realities of different production systems [12,15,16].

Despite the growing acknowledgment of the donkey’s economic significance, its evidence-based nutritional management is hindered by critical knowledge gaps. Current understanding is fragmented, often relying on unvalidated extrapolations from horse or ruminant studies. Furthermore, inconsistent methodologies across existing research prevent meaningful comparative analysis, and the mechanistic links between diet, gut microbiota, and production outcomes remain poorly defined.

This narrative review addresses these shortcomings by providing a comprehensive, qualitative synthesis of the field. Unlike systematic reviews, which follow rigid protocols such as PRISMA guidelines, this narrative review offers a flexible, critical examination of the literature without quantitative meta-analysis.

## 2. Literature Search Strategy

This narrative review was conducted to provide a comprehensive and critical synthesis of current knowledge on the interactions between dietary strategies, gut microbial communities, and physiological outcomes in donkeys. To achieve this aim, a broad and iterative literature search strategy was employed, designed to capture the multidisciplinary scope of the topic rather than to exhaustively identify every potentially relevant study as required for a systematic review. Search Strategy and Sources: Electronic searches were performed in major academic databases, including PubMed, Web of Science, Scopus, and Google Scholar. Search terms were combined using Boolean operators and included keywords related to the host (donkey, Equus asinus, equid), the intervention (nutrition, diet, feed, roughage, concentrate, protein, energy, supplementation), the mechanism (gut microbiota, microbiome, hindgut fermentation, cecum, colon), and the outcomes (growth, health, digestibility, antioxidant, inflammation). The search strategy was refined iteratively based on initial results. Retrieved records were initially screened by title and abstract to assess relevance to the core themes of donkey nutrition and gut microbiota. Full-text articles of potentially relevant studies were then obtained and evaluated. The synthesis is presented in a narrative format, integrating findings across studies to elucidate patterns, mechanisms, and future research directions.

## 3. Forage Studies on Donkeys

### 3.1. Foraging Behavior

**Figure 1:**
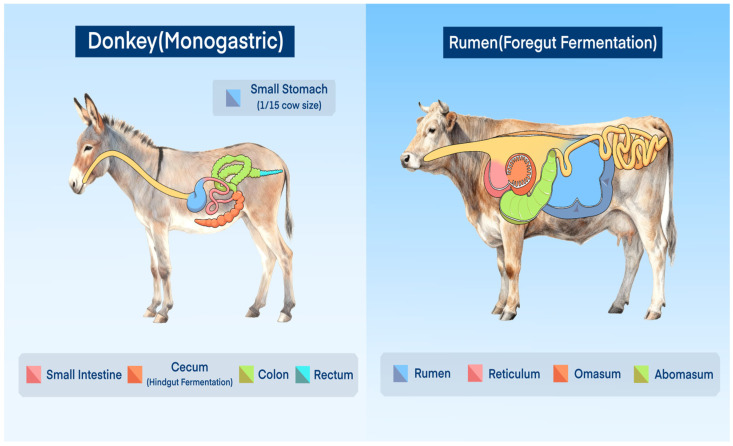
Donkey vs. ruminant digestive systems.

The modern donkey originates from the *Equus africanus* and belongs to the family *Equidae* of mammals [27]. Donkeys primarily consume high-fiber, low-energy diets, and their nutritional requirements for energy and protein are significantly lower than those of horses. Unlike other herbivores such as cattle and sheep, donkeys are non-ruminant, single-stomached herbivores and do not possess a rumination mechanism. Moreover, the stomach capacity of a donkey is relatively small, amounting to only about one-fifteenth that of a cow of comparable body size. Donkeys possess a highly developed cecum, which plays a central role in the digestion and absorption of nutrients. Due to physiological differences from ruminant animals, donkeys exhibit distinct feeding behaviors compared to other herbivores. Both donkeys and horses are non-ruminant herbivores, relying on hindgut fermentation to digest dietary fiber, whereas ruminants such as cattle and water buffalo utilize foregut fermentation in the rumen to break down fiber. This fundamental difference means that non-ruminants have less opportunity to absorb the end products of fiber digestion compared to ruminants are shown in Figure 1.

**Figure 2:**
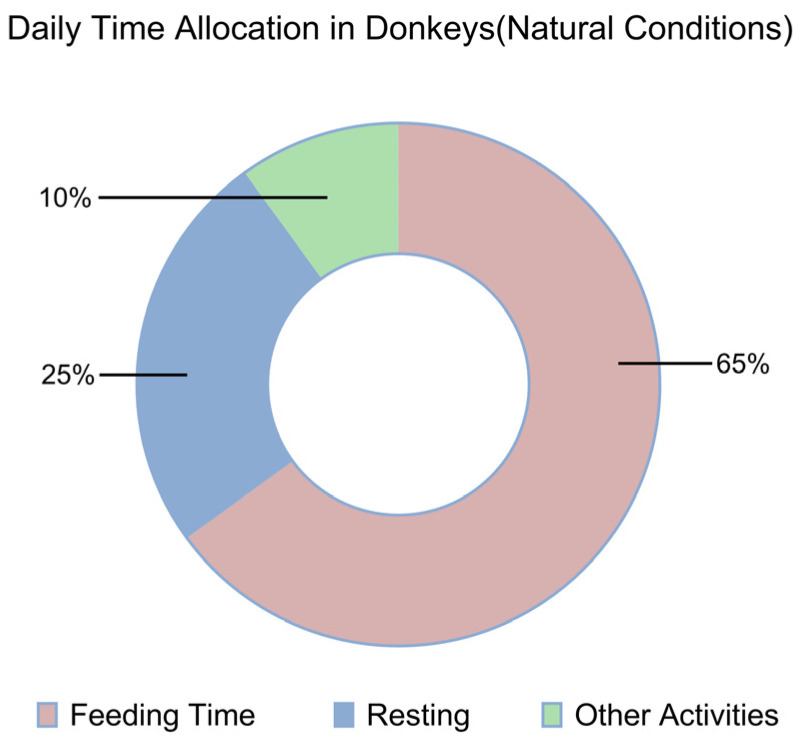
Donkey feeding behavior characteristics.

Given adequate feeding time, ruminants such as cattle can consume dry matter equivalent to approximately 2% of their body weight per day when fed the same type of roughage. In comparison, donkeys typically consume dry matter amounting to 2.0–2.5% of their body weight, while large horses may consume 2.5–3%. Overall, donkeys consume about 25–30% less feed than horses. However, donkeys exhibit higher digestive efficiency for low-quality roughages compared to horses. When fed highly digestible, high-quality forages such as alfalfa, the difference in nutrient utilization between donkeys and horses is minimal. In contrast, when fed low-quality forages such as corn stalks or barley straw, donkeys demonstrate up to 30% higher fiber digestibility than horses. The underlying reasons for these differences are not yet fully understood; however, two factors have been well established through research. First, there are notable differences in the microbial populations present in the hindgut. Second, donkeys have a proportionally larger gastrointestinal tract relative to body volume, which enhances their ability to digest fibrous forages. Traditionally, the belief that donkeys are “easier to feed” than horses was attributed to their lower dietary intake requirements. In fact, there is no substantial quantitative difference between donkeys and horses in terms of their energy and protein requirements. Under natural or grazing conditions, donkeys typically spend the entire day and part of the night—usually between 14 and 16 h—searching for and consuming feed are shown in Figure 2 [27]. Donkeys have higher feed selection requirements, and when suitable feed is not found, they typically continue searching for appropriate forage. Research has demonstrated that equids tend to prefer stems over leaves, meaning they select for fiber. Observations on the foraging behaviors of Burchell’s zebra and the wildebeest showed similar preferences for fibrous materials. Equids, compared to ruminants, tend to select more stems as feed [27]. Since donkeys lack a rumination mechanism, restricting their feeding time results in an increase in their digestive coefficient and a slower average retention time compared to ad libitum feeding. Additionally, donkeys have a high nitrogen recycling capacity [12]. The donkeys spend more time chewing and have longer feeding durations. They feed slowly and exhibit strong adaptability to different types of forage. Under natural conditions, donkeys can meet their nutritional needs by consuming low-quality, fibrous roughage, with feeding durations lasting 14 to 17 h [28].

### 3.2. Roughage for Donkeys

**Figure 3:**
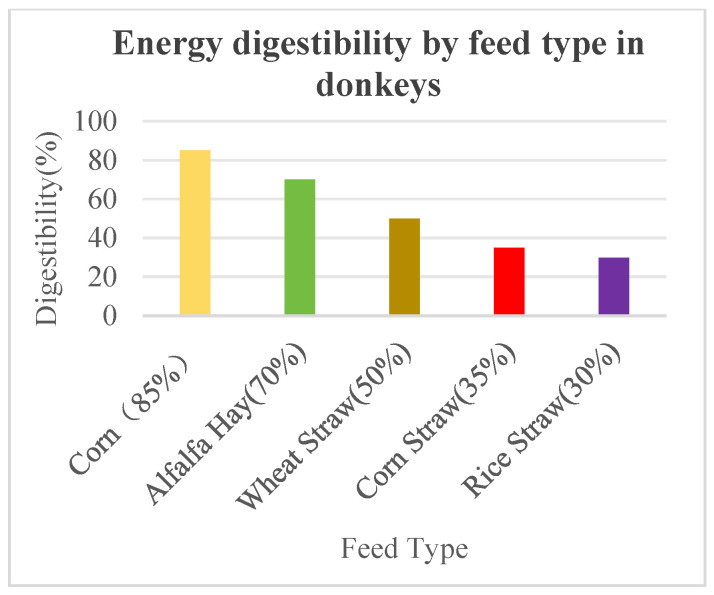
Feed categories and digestibility for donkeys.

Roughage is a primary feed source for donkeys and plays a crucial role in donkey husbandry [29]. The production and utilization of roughage are essential for ensuring the health and performance of donkeys. Common types of roughage in donkey husbandry include fresh grass and crop residues [30]. Roughage is characterized by its high crude fiber content and relatively low digestibility. The crude fiber content of roughage generally ranges from 25% to 50%, with hay typically containing lower levels around 25% to 30%. The digestibility of organic matter in roughage is generally below 70%. Despite its high crude fiber content and low nutritional value, roughage remains vital for herbivorous livestock, especially in winter when it becomes the primary feed. While its nutritional value is low in terms of energy, it helps maintain livestock nutrition [31]. The digestibility of different feeds by donkeys is shown in Figure 3 [32]. The protein content in roughage varies considerably due to the wide range of feed types, preparation methods, and other factors [33]. For instance, legume hays contain 10% to 19% crude protein, while grass hays contain 6% to 10%. In contrast, the crude protein content in grass straw and chaff is only 3% to 5%. The digestibility of protein is also higher in legume hay than in straws and chaff, with alfalfa hay exhibiting a protein digestibility rate of 71%, compared to 50% for grass hay and 15% to 20% for straws. Regarding mineral content, roughage generally has a higher calcium content, with legumes and straw containing around 1.5% calcium, while grass hays and straw contain only 0.2% to 0.4%. Phosphorus content in roughage is generally low, ranging from 0.1% to 0.3%, with straw-based feeds containing less than 0.1% phosphorus. The vitamin content in roughage is also variable, with high-quality hay, especially legume hay, containing substantial amounts of carotene and vitamin D, whereas most straws and chaff are nearly devoid of carotene and B vitamins.

#### 3.2.1. Conventional Roughage on Donkeys

The range of feed types used for donkeys is quite broad. Compared to horses, donkeys have relatively lower requirements for forage quality, and most crop residues can be used as conventional roughage for donkeys. The sources of roughage for donkeys are diverse, including hay, straw, and chaff. Straw-based roughage is high in crude fiber, coarse in texture, and low in digestibility. The fiber is primarily concentrated in the cell walls, which account for more than 70% of the content. These fibers can be broken down by gut microorganisms into volatile fatty acids. Straw has very low crude protein and vitamin content, making it nutritionally incomplete. The maturity of straw somewhat affects its digestibility; the more mature the straw, the higher its lignification, and the lower its digestibility. Common types of straw roughage include corn stalks [34], wheat straw [35], rice straw, bean straw [36], sorghum stalks [37], and oat straw [38]. Corn stalks contain over 30% carbohydrates, 2–4% protein, and 0.5–1% fat. Due to their coarse texture, corn stalks need to be processed into fine, chopped material for feeding donkeys. If simply chopped, they are less palatable for donkeys. After microbial fermentation, however, they can be used as silage, either green or dry, which improves palatability. Corn silage is highly nutritious, with a mild fruity aroma after fermentation, and is juicy, which enhances its palatability [39]. Feeding a moderate amount of corn silage can promote digestion in donkeys [40]. However, since donkeys are physiologically more suited to neutral or slightly alkaline feeds, corn silage, being acidic, should not be fed in excess. Wheat straw is a byproduct of millet crops and has higher dry matter digestibility compared to wheat and rice straw. It is one of the commonly used roughages in northern China.

#### 3.2.2. Locally Sourced Non-Conventional Roughage on Donkeys

Donkeys have a strong ability to digest roughage, and the feed resources vary across different regions. As a result, the types of roughage fed to donkeys’ change depending on the location and season. In the eastern part of Shandong Province, where rivers are dense and water resources are abundant, reed grows lushly. Fresh reeds can be cut and chopped and directly fed to donkeys. Alternatively, collected reeds can be fermented and made into reed silage [41], significantly reducing the cost of donkey husbandry. Garlic skins and garlic stems, byproducts of agricultural processing, have multiple bioactive properties [42]. However, high doses of organosulfur compounds may inhibit fiber-fermenting bacteria. Garlic products are widely used as natural additives in animal production as alternatives to antibiotics [43,44,45]. Organic sulfur compounds such as allicin, found in garlic products, have the potential to inhibit the synthesis of membrane lipids in prokaryotic communities, thereby reducing the population of methane-producing microbes and decreasing methane emissions [42,46].Bamboo leaves have potential applications in donkey feed, especially in areas with drought or limited forage resources [47]. The use of bamboo leaves in feed offers both research and practical value, particularly in sustainable livestock farming and the diversification of feed resources [48]. Bamboo leaves are rich in crude fiber, meeting the structural feed requirements of donkeys. Compared to straw-based feeds, bamboo leaves have a higher nitrogen content. Furthermore, bamboo leaves contain antioxidant compounds [49,50,51], which can help enhance animal immunity and stress resistance [52]. They are suitable for use as a supplement during dry seasons or times of forage scarcity. Bamboo leaves can be sun-dried, crushed into powder, and added to compound feeds. They can also be fermented with microorganisms (such as lactic acid bacteria [53] to improve palatability and digestibility. Additionally, they can be mixed with other green forages (e.g., corn stalks) for silage [54], improving nutritional balance.

### 3.3. Concentrated Feed for Donkeys

Concentrated feed for donkeys refers to feeds with high energy and protein content, small volume, and high digestibility, in contrast to roughages such as grass, straw, and bamboo leaves. It is a key component for improving the production performance of donkeys [55]. The primary role of concentrated feed is to provide high energy, supporting physiological activities such as physical labor, growth, fattening, and reproduction. Providing and supplementing protein in the diet promotes muscle development, coat brightness, and healthy growth [56]. Additionally, feeding concentrated feed plays a significant role in shortening the fattening period, enhancing immunity, and increasing feed intake and daily weight gain [57].Common concentrated feed ingredients can be categorized by function into: energy feeds, protein feeds, bran and husk feeds, and oilseed feeds. Energy feeds mainly include raw materials such as corn, sorghum, and wheat. These concentrated feeds are high in carbohydrates and provide energy to sustain the donkey’s physical activity. Protein feeds mainly include high-nitrogen ingredients such as soybean meal, cottonseed meal [58], and rapeseed meal. Due to their high protein content, these feeds have a positive impact on the growth and development of donkeys. Bran and rice bran are examples of bran and husk feeds [59]. These materials promote digestion and enhance palatability during the donkey’s feeding and digestion processes while also supplementing energy and crude fiber. Oilseed feeds, when added in small quantities, increase the energy density of the diet [60].Comparison of Different Roughages are shown in Table 1. It is important to note that concentrated feeds cannot completely replace roughages during feeding. Donkeys require sufficient fiber to maintain microbial health in the stomach, cecum, and colon. In the use of concentrated feeds, it is essential to maintain dietary balance and ensure a proper ratio of energy to protein to avoid feed waste and nutritional imbalances.

## 4. Nutritional Studies on Donkeys

### 4.1. Energy Requirements

The nutritional requirements for donkeys have been summarized in Table 2. Donkeys are non-ruminant animals, and their primary sources of energy, like other animals, include corn, oils, wheat, and their by-products. Insufficient energy intake can lead to weight loss, delayed estrous cycles in female donkeys, reduced semen quality in male donkeys, and restricted growth of donkey foals [64]. Studies have shown that adding hemp oil to feed can effectively improve the sperm quality of male donkeys and enhance their reproductive capacity [65]. Conversely, excessive energy intake can cause obesity, toxemia (such as laminitis), and a decrease in reproductive performance and service life of female donkeys. Research has shown that the basal metabolic rate of donkeys is 20% lower than that of horses, and donkeys can also adjust their basal metabolic rate based on the quality of the diet. Due to their smaller body size, donkeys consume less feed during physical work compared to horses and cattle. Donkeys primarily obtain energy from carbohydrates, including starch and fiber, which are main structural carbohydrates. Feeds from grains (corn, barley, and wheat) are rich in starch, which is hydrolyzed into glucose. Fibrous feeds are rich in structural carbohydrates such as cellulose, which, after digestive hydrolysis, produce volatile fatty acids [66,67]. Both glucose and volatile fatty acids provide energy for donkeys [68]. In addition to carbohydrates, donkeys can also use dietary fats and proteins as energy sources. When energy intake exceeds the body’s needs, the excess is converted into fat. To prevent energy deficiency or excess, understanding the energy requirements of donkeys is crucial. Carbohydrates are the most abundant nutrients in feed and consist of two main components: non-nitrogen extract [69] and crude fiber [70]. Non-nitrogen extract includes monosaccharides, disaccharides, and starch, which are easily digestible and are the primary source of energy for livestock. Enzymes in the salivary glands can break down a small amount of starch, and starch is further broken down into monosaccharides in the intestines by amylase, which are absorbed [71]. Some of the monosaccharides are directly oxidized to provide energy for maintaining body temperature and muscle function, while others are converted into glycogen stored in the liver or muscles. The remainder is converted into fat and stored in the body [72]. Crude fiber contains cellulose, hemicellulose, lignin, and other substances, which are difficult to digest, but are essential for herbivorous livestock. For donkeys, crude fiber is primarily digested and fermented by microorganisms in the cecum and colon, where it is converted into volatile fatty acids such as acetic acid, propionic acid, and lactic acid. These fatty acids are absorbed and used as energy [67]. Additionally, crude fiber plays a role in filling the digestive tract, providing satiety and hunger resistance [73], and mechanically stimulating the gastrointestinal tract to promote peristalsis and increase digestive fluid secretion, which benefits feed digestion and fecal excretion [74]. Therefore, it is essential to provide donkeys with sufficient fiber-containing feed. The digestibility of carbohydrate feed for donkeys depends on the nature of the feed. For example, donkeys can digest 85% of the energy in corn kernels, but only 35% of the energy in corn stalks. This is because corn stalks are high in crude fiber, with minimal energy being utilized, and most of the energy is excreted in feces. Therefore, the energy requirement for both horses and donkeys is generally defined as the amount of digestible energy required. A donkey weighing 150 kg has a daily requirement of 20 MJ of digestible energy, which should be correlated with the actual feed intake. However, it should be noted that studies have shown that compared with high-fiber diets, high-starch diets are more likely to affect the microbial stability in the equine gut and increase the risk of disease. This issue should also be taken into account in the dietary management of donkeys [75].

### 4.2. Protein Requirements

Proteins are broken down in the stomach by pepsin and further digested in the intestine by pancreatic and intestinal proteases, gradually decomposing into amino acids, which are absorbed and utilized by the body. Excess protein can be converted into fat and stored in the body. When necessary, protein can also serve as a source of energy. Proteins are composed of polypeptide chains formed by the linkage of individual amino acids. The properties of a protein are determined by the types and numbers of amino acids that make up the polypeptide chain [76]. There are 20 common amino acids, some of which can be synthesized by donkeys’ bodies and do not need to be supplied through the diet; these are known as non-essential amino acids. Others cannot be synthesized or cannot be synthesized in sufficient quantities to meet the body’s growth or production needs and must therefore be obtained through the diet; these are called essential amino acids (such as methionine, lysine, etc.). If the diet lacks essential amino acids (such as lysine) [77], it impairs the growth and development of young ruminants, which in turn leads to dry skin, poor coat quality, reduced appetite, and weakened physical condition. Unlike ruminants, equids cannot efficiently absorb amino acids synthesized by microorganisms—this is because such synthesis occurs after the small intestine, resulting in microbial-synthesized essential amino acids contributing minimally to donkeys [78]. Various feedstuffs contain certain amounts of protein [79]. Generally, legumes contain more protein than grasses [80]. Within the same plant, the leaves and branches contain more protein than the stems; the protein content is higher during the early growth stages and decreases rapidly during flowering; the highest protein content is found in the seeds after the plant matures, with the stems containing the least. Animal-derived feeds are rich in protein and of high quality. Young donkeys, due to their rapid growth, require more protein supplementation. Other donkeys, however, cannot store excess amino acids in their bodies, so their daily protein requirements must be met through feed. However, feeding excessive protein is also wasteful. Ruminants can supplement nitrogen deficiency by utilizing urea [81], but feeding urea to donkeys leads to direct absorption through the stomach wall and intestinal mucosa into the bloodstream, without interaction with colonic microbes, which can cause urea poisoning and even death in donkeys. Therefore, urea should not be fed to donkeys [82]. In ruminants, another pathway for obtaining essential amino acids (EAA) is the absorption of microbial protein produced during the digestion of fiber [83]. Donkeys also have a similar digestive process, although these microbially produced EAAs cannot be digested and absorbed before excretion. It has been observed that in many tropical countries, donkeys fed low-protein diets can still grow normally, indicating that the protein digestion and metabolism processes in donkeys are more complex.

### 4.3. Vitamin and Mineral Requirements

Vitamins and minerals play a crucial role in the growth, reproduction, and overall health of donkeys [84]. Minerals are especially important for the growth and development of foals, as well as the health and reproduction of adult donkeys. They are not only essential components of donkey body tissues but also play an indispensable role in regulating body fluid pH and maintaining osmotic pressure. The minerals required by donkeys are categorized into major and trace elements. Major elements include calcium, potassium, chlorine, sodium, phosphorus, magnesium, and sulfur, while trace elements include iron, copper, manganese, zinc, iodine, cobalt, selenium, molybdenum, fluorine, and chromium. Under normal conditions, donkeys can effectively absorb vitamins C and D, but they cannot synthesize vitamins A and D. Vitamins A, E, K, and B complex are particularly prone to deficiency in donkeys, and proper supplementation should be considered during management. However, most existing studies on the vitamin and mineral requirements of donkeys refer to the needs of small horses as outlined by the NRC (National Research Council), with the recommended intake being approximately 75% of that for small horses of equivalent weight. More research is needed to accurately determine the specific requirements of donkeys for vitamins and minerals [85]. Calcium and phosphorus are the most important minerals [86], comprising approximately 70% of the total mineral content in the body. Ninety-nine percent of calcium and over 80% of phosphorus are stored in the bones and teeth. Calcium plays a significant role in bone growth, regulating fluid balance, and blood coagulation [87]. Donkeys absorb calcium and phosphorus from feed, but they can only digest, absorb, and utilize these minerals effectively if the calcium-to-phosphorus ratio is appropriate (1.5:1 or 2:1) and there is adequate vitamin D supply. Legumes, such as alfalfa, and certain grasses and hay, contain higher levels of calcium, whereas grasses and straw from the *Poaceae* family generally have lower calcium and phosphorus content. Seed grains and their byproducts typically contain more phosphorus than calcium. Donkey diets are primarily based on green roughage with a certain number of concentrates, so deficiencies in calcium and phosphorus are rare. However, foals in the growth stage, pregnant and lactating females, and breeding males require more calcium and phosphorus. Diets with higher amounts of concentrate often result in an imbalance, with excess phosphorus and insufficient calcium. Therefore, it is essential to supplement the diets of breeding males, pregnant females, and foals with calcium-rich feed [88]. Sodium, chlorine, and potassium are physiologically important [89]. They maintain the acid-base balance in the body, regulate the osmotic pressure between cells and blood, ensure that body tissues retain an adequate amount of water, and help regulate cardiac function. Chlorine is a precursor in the production of gastric hydrochloric acid and is abundant in milk [90]. A deficiency in chlorine and sodium often results in dehydration, poor digestion, reduced appetite, rough and dry coat, malnutrition, and decreased productivity in donkeys. Therefore, it is essential to provide adequate salt (sodium chloride) to donkeys to meet their chlorine and sodium requirements [91]. Providing salt blocks is more effective than adding salt directly to the diet, as excessive salt in feed can suppress appetite and reduce overall nutrient intake. In practical feeding, it is also important to understand the acid-base balance of the diet. Acid-base feed refers to the ratio of acidic elements (such as sulfur, phosphorus, and chlorine) to alkaline elements (such as sodium, potassium, calcium, and magnesium) in the feed. Generally, roughage, silage, and green forage are considered physiologically alkaline, while concentrates, such as sorghum, corn, soybeans, soybean meal, wheat, and bran, are considered physiologically acidic. However, millet and similar grains are considered mildly alkaline feed. For the normal physiological needs of livestock, a neutral or slightly alkaline diet is preferable.

### 4.4. Water Supply Requirements

Water is a major component of the donkey’s body and plays a crucial role in maintaining normal physiological functions. Approximately 60% of a donkey’s body weight is water, 82% of its blood consists of water, and even the bones contain 25% water. Donkeys are well-adapted to semi-arid environments and can manage thirst effectively, quickly replenishing fluids when needed. They can tolerate water loss up to 30% of their body weight and, within 2 to 5 min, drink 24 to 30 L of water to rapidly rehydrate and recover. Donkeys are more resilient to hunger and thirst compared to ponies, and they can consume feed with little water for extended periods [92]. However, it is important to distinguish between their short-term ability to endure thirst and their long-term water requirements [12]. Various factors, such as temperature, season, diet composition, feeding level, and drinking intervals, can all influence the amount of water donkeys consume. Under normal circumstances, depending on changes in environmental factors, the water requirement of donkeys for maintaining their metabolic levels is generally 35–95 g/kg·BW^−1^ [93,94]. During hot seasons, their water intake may be twice as much as in cooler seasons. Lactating donkeys require a significantly higher water intake to support milk production, approximately double the amount needed by non-lactating donkeys. When temperatures exceed 30 °C, water consumption can increase to 3 to 4 times the usual amount. Overall, the water requirements of donkeys are similar to those of horses.

**Table 2:** Nutritional requirements summary for donkeys.

Nutrient Category	Requirement	Key Notes	Comparison to Horses
Energy (digestible)	20 MJ/day (150 kg donkey)	20% lower basal metabolic rate	Lower requirements
Dry matter intake	2.0–2.5% body weight	25–30% less than horses	Lower intake
Protein	Stage-specific needs	Essential amino acids critical	Similar quality needs
Crude fiber	25–50% of diet	Higher digestibility than horses	30% better utilization
Water	35–95 g/kg·BW^−1^	Can tolerate 30% water loss	Similar to horses
Ca: P	1.5:1 to 2:1 ratio	Vitamin D required for absorption	Similar ratios

The information provided in Table 2 are adopted from previous published data [12,16,95,96,97,98].

## 5. The Impact of Donkey Intestinal Microbiota on Donkey Growth

In general, the richness and diversity of the microbiota are much higher in the hindgut relative to that in the foregut; at phylum level, the Firmicutes is dominant in the foregut while both Firmicutes and Bacteroides are abundant in the hindgut; at the genus level, Lactobacillus was dominant in the foregut while Streptococcus was more dominant in the hindgut [99]. The diversity and richness of hindgut microbiota are extremely significantly higher than those of foregut microbiota, with no gender differences. The community structure and composition in the same or adjacent regions are similar. There are also differences in the composition of intestinal microbial communities among donkeys from different geographical regions; for example, the diversity and richness of intestinal microbiota in Qinghai donkeys from the Qinghai–Tibet Plateau are significantly higher than those in Dezhou donkeys [100]. However, donkeys themselves do not possess enzymes that can directly degrade cellulose in the cell walls of feed. They mainly rely on microorganisms in the cecum and colon to digest roughage. For instance, anaerobic fungi can invade the fibrous tissues in the feed cell walls through their mycelial rhizoids and secrete a series of highly active degrading enzymes to efficiently break down feed fibers. The volatile fatty acids (VFAs) produced by the degradation of fibrous feed by the microorganisms in the hindgut of donkeys can provide approximately 60–70% of the metabolic energy for the body [101]. Donkey intestinal microbiota can also affect the metabolism of nutrients. Different feeding sequences of concentrated feeds will influence the composition and abundance of donkey intestinal microbiota, thereby affecting the digestion and metabolism of nutrients. This study shows that in the group where energy feeds and protein feeds are fed simultaneously, the digestibility of crude protein and crude fat in donkey foals is higher [102]. This may be because a reasonable feeding sequence optimizes the structure of intestinal microbiota, promotes the growth of beneficial flora, and thus enhances the ability to digest and absorb nutrients. Optimizing the feeding sequence of concentrated feeds can improve the structure of intestinal microbiota, thereby enhancing the immune system of donkey foals and increasing their disease resistance. A healthy intestinal microbiota structure helps maintain the integrity of the intestinal mucosal barrier, reduces the invasion of pathogens, lowers the risk of disease occurrence, and thus promotes the growth and development of donkeys [103]. By mitigating pathogen invasion and lowering disease risk, a healthy microbiota ensures that donkeys maintain a stable metabolic state conducive to growth. Pathogenic infections, conversely, disrupt normal physiological processes: they trigger immune responses that divert significant energy and nutrients toward fever, inflammation, and tissue repair—resources that would otherwise be allocated to muscle development, bone growth, or fat deposition. For example, bacterial enteritis can impair nutrient absorption by damaging intestinal villi, leading to malabsorption and weight loss, while systemic infections may suppress appetite and increase metabolic demands, resulting in a negative energy balance. In severe cases, chronic or recurrent diseases can lead to stunted growth, as the body prioritizes survival over growth-related processes [104].

Thus, the intestinal microbiota’s positive regulation of the immune system operates as an indirect but critical driver of donkey growth. By sustaining barrier integrity, modulating immune function, and reducing disease burden, a balanced microbial community creates an optimal physiological environment for efficient nutrient utilization and sustained growth.

## 6. Nutritional Intervention and Its Effect on Donkey Microbiota

The normal microbiota composition across various segments of the donkey gastrointestinal tract has been comprehensively characterized in our previously published research [105]. Additionally, we demonstrated how alterations in microbiota composition may influence donkey health and production outcomes. The gastrointestinal microbiota of donkeys plays a crucial role in nutrient metabolism, immune function, and overall health, making it a key target for understanding how dietary modifications can optimize animal welfare and performance.

### 6.1. Mechanistic Pathways Linking Diet, Microbiota, and Health Outcomes in Donkeys

Understanding the mechanistic basis of how dietary interventions modulate gut microbiota to influence donkey health requires integration of nutritional biochemistry, microbial ecology, and host physiology. This section synthesizes current evidence to establish causal pathways from nutritional modifications through microbiota alterations to measurable health and production outcomes, representing a novel framework for donkey nutrition research.

#### 6.1.1. Protein-Microbiota-Growth Performance Axis

Dietary protein supplementation fundamentally alters the hindgut microbial ecosystem through multiple mechanisms. When dietary protein increases to optimal levels (12.52% crude protein), the enhanced substrate availability for proteolytic bacteria promotes selective proliferation of beneficial genera including *Prevotella*, *Ruminococcus*, and *Bacteroides* [106,107]. These bacteria possess specialized enzymatic machinery for protein degradation, producing bioactive peptides and amino acids that may be absorbed and utilized for muscle protein synthesis, potentially being associated with growth performance improvements observed in preliminary studies [77,108]. While protein-derived amino acids serve as precursors for microbial synthesis of B vitamins and essential cofactors, their contribution to host metabolic efficiency in equids remains to be validated [81]. Observational studies have found that increased abundance of Prevotella is associated with enhanced production of branched-chain amino acids (BCAAs); however, the assumption that hindgut-produced BCAAs stimulate mammalian target of rapamycin (mTOR) signaling pathways in donkeys is based on extrapolation from rodent models and requires validation, as BCAAs produced in the cecum/colon are not efficiently absorbed into systemic circulation [108]. These mechanistic pathways, while plausible, have not been directly demonstrated in equids and require integrated metabolomics and mucosal biopsy studies for confirmation.

This protein-microbiota-growth axis demonstrates that nutritional interventions do not simply provide nutrients directly to the host but rather modulate the microbial community to create a synergistic environment that amplifies nutrient utilization efficiency. The observed improvements in nutrient digestibility following protein supplementation [107] can be mechanistically attributed to microbiota-mediated enhancement of intestinal barrier function. Beneficial bacteria such as *Akkermansia muciniphila*, which increased significantly in response to protein supplementation [109]. However, *Akkermansia* may excessively degrade mucin, which could instead increase intestinal permeability; its beneficial effects are species-specific and context-dependent. The produced metabolites may have the effect of enhancing the tight junction proteins in the intestinal epithelium, thereby reducing intestinal permeability and improving nutrient absorption, while also being associated with preventing the translocation of pathogenic bacteria and their endotoxins [110]. This represents a critical mechanism linking dietary protein → microbiota modulation → intestinal health → improved nutrient utilization and growth performance. It should be noted that *Akkermansia muciniphila*’s role in equids may be context-dependent and not inherently beneficial. While often associated with improved metabolic health in other species, excessive mucin degradation can potentially thin the protective mucus layer, and its effects are strongly influenced by dietary fiber characteristics. Donkey- and equine-specific research on *Akkermansia* remains limited, necessitating cautious interpretation of its functional significance.

#### 6.1.2. Methionine Supplementation and the Oxidative Stress-Antioxidant-Microbiota Triangle

Methionine supplementation reveals a sophisticated bidirectional relationship between dietary amino acids, gut microbiota, and systemic antioxidant capacity. At supplementation levels of 5–15 g/day, methionine significantly enhanced antioxidant enzyme activities (CAT, T-SOD, GSH-Px) while reducing lipid peroxidation markers (MDA) [111]. The mechanistic pathway operates through multiple nodes. First, methionine serves as a precursor for glutathione synthesis, the cell’s primary antioxidant defense molecule [112]. Second, methionine supplementation selectively promoted growth of *Methanocorpusculum* and *Ruminococcus* species [111], which produce short-chain fatty acids (SCFAs), particularly butyrate, that are associated with potential upregulation of host antioxidant enzyme gene expression through histone deacetylase (HDAC) inhibition and nuclear factor erythroid 2-related factor 2 (Nrf2) pathway activation [113,114]. This creates a synergistic cycle where dietary methionine → increased beneficial microbiota → enhanced SCFA production → upregulated host antioxidant systems → reduced oxidative stress → improved gut barrier integrity → healthier microbiota ecosystem. The bidirectional nature is critical; while methionine modulates microbiota composition, the resulting microbial metabolites (particularly butyrate and propionate) further enhance the host’s capacity to utilize methionine for antioxidant defense [115]. This triangle represents a novel understanding that amino acid supplementation effects extend far beyond simple substrate provision, instead orchestrating complex microbiota-metabolite-host signaling cascades. However, there are currently no mechanistic studies or validations targeting donkeys. In the future, it will be necessary to combine integrated metabolomics with mucosal biopsies to verify these pathways.

#### 6.1.3. Energy Optimization and the Inflammation-Microbiota-Immunity Nexus

Energy level manipulation provides perhaps the most dramatic demonstration of diet-microbiota-health interactions in donkeys. However, energy optimization in hindgut fermenters like donkeys requires careful consideration of starch overload risks. High-energy diets typically rely on increased starch content, but excess starch bypassing small intestine digestion undergoes rapid fermentation in the cecum, leading to pH reduction and subclinical or clinical hindgut acidosis. This disrupts cellulolytic bacteria populations and promotes acid-tolerant species like Lactobacillus and Streptococcus. The resulting endotoxin release can trigger systemic inflammation and increase laminitis risk, which are fundamental physiological principles in equid nutrition that must be considered when optimizing energy levels. Medium energy levels (10.49 MJ/kg) during the periparturient period significantly modulated both pro-inflammatory and anti-inflammatory pathways [24]. The mechanistic cascade begins with energy substrate availability influencing microbial fermentation patterns whereby optimal energy levels promote saccharolytic fermentation by beneficial genera (*Candidatus_Saccharimonas*, *Fibrobacter*, *Lactobacillus*, *Bifidobacterium*, *Akkermansia*), which produce SCFAs with potent anti-inflammatory properties [24,116]. Based on rodent studies, butyrate has been shown to activate G-protein coupled receptors (GPR43/GPR109A) on intestinal epithelial cells and immune cells, potentially triggering anti-inflammatory signaling cascades that may reduce production of pro-inflammatory cytokines (TNF-α, IL-1β, IL-6) [117,118]. However, the expression and function of these receptors in donkey tissues have not been characterized, and the systemic endocrine effects of hindgut-produced SCFAs in equids remain largely unstudied. Although short-chain fatty acids (SCFAs) can act locally on the intestinal epithelium, research on their systemic endocrine effects in equids remains quite limited. Studies on butyrate receptors (GPR41/43) have been conducted exclusively in rodents and have not been characterized in equine species. Furthermore, the hypothesis that SCFAs produced in the colon can significantly regulate systemic inflammation has not yet been confirmed. Conversely, excessive energy supplementation promotes proliferation of bacteria associated with inflammatory responses (*norank_f_norank_o_Mollicutes_RF39*, *norank_f_norank_o_Coriobacteriales*), which produce metabolites that activate toll-like receptors (TLRs) on innate immune cells, triggering nuclear factor kappa B (NF-κB) signaling and inflammatory cytokine production [24,119]. This energy-microbiota-inflammation axis is particularly critical during the periparturient period when donkeys experience heightened oxidative stress and immune challenges [120]. Short-term studies have reported reductions in inflammatory markers (30–50% decreases in IL-1, IL-2, IL-6, TNF-α) following optimal energy supplementation [24] which may be associated with increased beneficial bacteria that are capable of producing anti-inflammatory SCFAs; however, whether these SCFAs achieve sufficient systemic concentrations to directly modulate inflammatory responses in donkeys remains unestablished [121]. While these associations suggest potential microbiota-mediated mechanisms, most evidence supporting SCFA-mediated immune modulation derives from rodent models, and the causal relationships in equids require validation through targeted mechanistic studies. Additionally, current donkey microbiota studies predominantly utilize fecal samples, which may not accurately reflect the mucosa-associated microbial communities where such host-microbe interactions would occur. This nexus reveals that energy nutrition does not simply provide metabolic fuel but rather determines the inflammatory tone of the entire organism through microbiota-mediated mechanisms, representing a paradigm shift from traditional energy nutrition concepts focused solely on metabolizable energy supply.

#### 6.1.4. Non-Conventional Feeds and Bioactive Compound-Microbiota Interactions

The utilization of non-conventional feeds (reed silage, bamboo leaves, garlic byproducts) introduces an additional layer of complexity through phytochemical-microbiota interactions. Garlic-derived organosulfur compounds, particularly allicin, exert selective antimicrobial effects, inhibiting methanogens and certain pathogenic bacteria while preserving or enhancing beneficial fermentative bacteria [41,45]. This selective pressure reshapes the microbial community toward a composition favoring efficient feed fermentation and reduced methane production, thereby improving energy retention [43,44]. Bamboo leaves contain polyphenolic compounds with prebiotic effects, selectively promoting growth of polyphenol-degrading bacteria that produce bioactive metabolites with antioxidant and anti-inflammatory properties [48,49,50]. These phytochemical-microbiota interactions represent an underexplored frontier in donkey nutrition, where the bioactive components of feeds may be equally or more important than their macronutrient composition in determining health outcomes through microbiota modulation.

### 6.2. Comparative Analysis and Pattern Recognition Across Nutritional Interventions

Comparative analysis of multiple intervention studies reveals consistent patterns and principles that transcend individual experimental designs, establishing generalizable frameworks for donkey nutrition management. This synthesis represents novel insights not apparent from individual studies.

#### 6.2.1. Universal Microbiota Responders to Nutritional Optimization

Across diverse nutritional interventions (protein supplementation, methionine addition, energy optimization, prebiotic supplementation), certain bacterial taxa consistently emerge as “keystone responders” whose abundance correlates with improved health outcomes. Specifically, *Firmicutes* phylum members (particularly *Ruminococcus*, *Lactobacillus*, and *Oscillospiraceae* family members) consistently increased with optimal nutrition across multiple studies [24,25,99,107,122]. These bacteria are primary cellulose degraders and SCFA producers, directly contributing to energy harvest from fibrous feeds [17,18]. Their consistent positive response suggests they serve as indicators of nutritional adequacy and could potentially be used as biomarkers for feed quality assessment in donkeys. *Akkermansia muciniphila* emerged as a critical health-promoting bacterium across interventions, increasing with protein supplementation, optimal energy levels, and prebiotic addition [24,104,123]. *A. muciniphila*’s has been extensively studied in rodent models, where it has been associated with intestinal barrier integrity and potential modulation of host metabolism**; however, its role as a universal indicator of gut health is species-dependent, and in some contexts, excessive mucin degradation by *A. muciniphila* may potentially compromise barrier function [108]. While correlational studies in donkeys have observed positive associations with antioxidant capacity and inverse associations with inflammatory markers [24,106], these relationships do not establish causality, and the beneficial effects of *A. muciniphila* may be context- and species-specific, requiring validation in equids. *Prevotella* species showed consistent positive responses to increased dietary protein and fiber content [25,107,117], reflecting their specialized capacity for complex carbohydrate and protein degradation. Their abundance correlates with improved nutrient digestibility and growth performance, positioning them as key facilitators of nutritional efficiency in donkeys. Conversely, *Proteobacteria* phylum members generally increased under nutritional stress (low energy, inadequate protein, poor feed quality) and correlated with inflammatory responses and oxidative stress [99,109,124]. Their proliferation often signals dysbiosis and compromised gut health, serving as potential early warning indicators of nutritional inadequacy. This pattern recognition across studies enables development of microbial signatures for nutritional status assessment, representing a novel diagnostic approach for donkey nutrition management not previously articulated in the literature.

#### 6.2.2. Dose–Response Relationships and Threshold Effects

Analysis across studies reveals non-linear dose–response patterns for nutritional interventions, with clear threshold effects. However, excessive protein supplementation in equids presents significant risks related to putrefactive fermentation in the hindgut. When excess protein reaches the cecum and colon, it undergoes proteolytic fermentation leading to increased ammonia production, elevated branched-chain fatty acid concentrations, and formation of toxic compounds including phenols, indoles, and biogenic amines. This process elevates luminal pH, impairs fiber-digesting bacteria, and creates systemic toxicity requiring increased hepatic detoxification. Research suggests that crude protein levels of 12–13% appear optimal [104,105], with higher levels potentially compromising hindgut health and placing metabolic burden on the liver. This threshold represents a balance between providing adequate substrate for beneficial proteolytic bacteria while avoiding protein fermentation-associated production of potentially toxic metabolites (ammonia, biogenic amines). Energy supplementation demonstrates a clear optimum at 10.49 MJ/kg [24], with both insufficient (reduced beneficial bacteria, poor growth) and excessive energy (increased inflammatory-associated bacteria, metabolic stress) producing suboptimal outcomes. This inverted U-shaped response suggests that energy level determines the balance between saccharolytic (beneficial) and proteolytic (potentially harmful) fermentation in the hindgut. Methionine supplementation shows beneficial effects across a range of 5–15 g/day [111], with intermediate doses providing optimal antioxidant enhancement. This relatively wide effective range suggests methionine’s effects operate through multiple pathways with different dose–response kinetics, providing flexibility in practical supplementation strategies. These dose–response patterns reveal that optimal donkey nutrition requires precision rather than simply maximizing nutrient supply, representing a critical insight for developing evidence-based feeding recommendations.

#### 6.2.3. Temporal Dynamics and Critical Windows for Intervention

Emerging evidence suggests that the timing of nutritional interventions significantly impacts their effectiveness through life-stage-specific microbiota developmental windows. The early life suckling period (0–2 months) represents a critical window for microbiota establishment, with enzyme supplementation [99] and high-quality protein sources [106] having disproportionately large effects on microbiota maturation and long-term digestive capacity. Interventions during this period shape the foundational microbial community structure that persists into adulthood, suggesting early nutrition has lasting effects through microbiota programming. The weaning transition period (2–6 months) offers both risk (weaning stress-induced dysbiosis) and opportunity (increased plasticity for beneficial modulation). Feeding sequence optimization [96,100,101] and prebiotic supplementation [123] show particularly strong effects during this window, stabilizing the microbial community and preventing pathogen establishment during this vulnerable period. The periparturient period in pregnant and lactating donkeys shows heightened sensitivity to energy and protein optimization [24,123], with microbiota-mediated effects on oxidative stress and inflammation being particularly pronounced. This reflects the increased metabolic demands and immunological challenges of reproduction, where microbiota modulation provides critical support for maternal health and milk production. Recognition of these critical windows enables strategic timing of nutritional interventions for maximum efficacy, representing a temporal dimension of nutrition management not previously systematically addressed in donkey research.

### 6.3. From Mechanisms to Management: Translating Microbiota Knowledge into Practical Feeding Strategies

The mechanistic understanding and pattern recognition established above enable development of evidence-based, microbiota-informed feeding strategies for donkey production systems, representing a paradigm shift from empirical feeding practices to precision nutrition.

#### 6.3.1. Microbiota-Based Nutritional Optimization Framework

Traditional donkey feeding relies on meeting crude nutrient requirements (protein, energy, fiber) without considering how different feed ingredients and combinations influence microbiota composition and function. The evidence synthesized here enables a new framework where feeding decisions are guided by their predicted effects on microbiota and resulting health outcomes. Protein source selection should prioritize not just crude protein content but protein quality and fermentability characteristics that favor beneficial bacteria. Soybean meal demonstrated superior effects on gut microbiota compared to other protein sources [106], attributed to its optimal amino acid profile and moderate fermentation rate that promotes *Akkermansia* and *Oscillospiraceae* without excessive ammonia production. This suggests protein source evaluation should include microbiota modulation potential alongside traditional metrics like digestibility and amino acid balance. Energy level management requires balancing total digestible energy with fermentation pattern optimization. The clear optimum at 10.49 MJ/kg for periparturient donkeys [24] reflects not just metabolic energy supply but the energy level that promotes beneficial saccharolytic fermentation while minimizing inflammatory-associated proteolytic fermentation. Practical implementation requires formulating diets that provide adequate energy through high-quality, moderately fermentable carbohydrate sources (e.g., optimally processed grains, high-quality forages) rather than excessive starch or simple sugars that may disrupt hindgut fermentation. Fiber source diversification emerges as a critical strategy for maintaining microbiota diversity and resilience. Different fiber types (cellulose, hemicellulose, pectin) select for different bacterial populations [30,32], and combining conventional roughages (corn stover, wheat straw) with non-conventional fibrous feeds (reed silage, bamboo leaves) provides a broader substrate spectrum supporting microbial diversity. The consistent association between high *Firmicutes* abundance and feed efficiency [99,122] suggests that feeds promoting cellulolytic bacteria should form the foundation of donkey diets. Furthermore, the effects of different fiber types on microbiota depend on several factors including lignification degree, hemicellulose solubility, neutral detergent soluble fiber content (pectins), particle size, and passage rate through the cecum and colon. These structural characteristics directly determine which bacterial populations proliferate, which volatile fatty acids are produced, and the efficiency of fiber digestion. Understanding cell-wall structure differences between roughages helps explain why donkeys digest coarse fiber more efficiently than horses.

#### 6.3.2. Regional Adaptation and Sustainable Feed Resource Development

The successful utilization of non-conventional feeds (reed silage in eastern regions, bamboo leaves in southern regions, garlic byproducts in processing areas) [40,41,46] demonstrates that microbiota-based nutrition can be adapted to local feed availability while maintaining or enhancing production outcomes. This regional adaptation principle offers a pathway toward sustainable donkey production that reduces dependence on conventional feed resources and enhances economic viability. Region-specific feed evaluation should assess not just nutritional composition but effects on microbiota and production outcomes in local donkey populations. Geographic variation in donkey gut microbiota [98] may influence responses to local feeds, necessitating region-specific validation of feeding strategies. The higher microbial diversity in Qinghai plateau donkeys compared to Dezhou donkeys [98] suggests that high-altitude populations may have enhanced capacity to utilize diverse, low-quality feeds, providing valuable information for developing altitude-specific feeding recommendations. Waste-to-feed valorization through microbiota-informed processing can transform agricultural and processing byproducts into valuable donkey feeds. Fermentation of crop residues (corn stover, wheat straw) with selected microbial inoculants [33,38,39,61] improves nutritional value while introducing beneficial bacteria that may colonize the donkey gut. Similarly, processing of garlic industry byproducts into feeds with antimicrobial and prebiotic properties [41,43] represents a circular economy approach where waste materials become functional feeds that improve animal health through microbiota modulation. Seasonal feeding strategies can leverage temporal variation in feed availability and quality. The observed seasonal variation in digestible energy requirements [94] likely reflects both environmental conditions and seasonal changes in feed quality that alter microbiota fermentation efficiency. Developing season-specific feeding recommendations that account for feed quality-microbiota-energy efficiency relationships could significantly improve nutritional management and economic returns.

#### 6.3.3. Practical Implementation Challenges and Solutions

Natural variation in feed composition, particularly forages and agricultural byproducts, creates inconsistency in microbiota-mediated effects. Implementation of quality assessment protocols focused on parameters affecting fermentation dynamics (fiber fractions, protein degradability, anti-nutritional factors) and standardized processing methods can mitigate this variability. Economic constraints limit adoption of precision nutrition approaches in many production systems. Tiered implementation strategies address this challenge, wherein foundational principles (optimal protein-to-energy ratios, fiber diversity) can be applied with minimal resources, while sophisticated interventions (amino acid supplementation, prebiotic addition, microbiota monitoring) are reserved for high-value operations. Economic analyses indicate microbiota-optimized nutrition improves feed efficiency with 15–25% growth performance improvements [24,104,120], generating returns that justify incremental costs. Knowledge transfer remains critical, as microbiota concepts are unfamiliar to many producers. Simplified educational materials emphasizing observable outcomes (improved body condition, reduced disease incidence, enhanced milk yield) and extension programs demonstrating microbiota-informed feeding strategies effectively bridge the research-to-practice gap.

### 6.4. Evidence-Based Study Summaries

Thus, maintaining optimal bacterial populations through appropriate nutrition can simultaneously enhance antioxidant defenses and modulate immune responses which are critical factors compromising productive efficiency of equines. Consistently, a study documented that donkey foals receiving concentrate supplements with soybean meal significantly enhanced beneficial gut microbiota including *Akkermansia*, *Oscillospiraceae*, *Porphyromonas,* and *Streptococcus* [106]. Furthermore, they reported that this microbiota positively correlated with improved growth performance, serum hormones, and metabolites, demonstrating soybean meal’s superior effects on gut health. Dietary protein supplementation (12.52%) show potential to modulate hindgut microbiota in donkeys, with key differential bacterial genera including *Prevotella*, *Clostridiumsensustricto1*, *NK4A214group*, *OscillospiraceaeUCG-002*, and *OscillospiraceaeUCG-005* [107]. These genera were modulated by protein supplementation, which enhanced microbial community composition and improved nutrient digestibility and overall performance. Methionine (Met) supplementation in donkeys reduced oxidative stress (lower Malondialdehyde) and enhanced antioxidant capacity (higher Total Antioxidant Capacity and Catalase Activity) [109]. Furthermore, they revealed that Met (5 g/d) increased the abundance of *Methanocorpusculum* and *Ruminococcus*, while Met (15 g/d) altered gut microbiota including *Ruminococcus*, *Peptococcus,* and *Anaeroplasma*. These beneficial bacteria were positively associated with enhanced antioxidant activity and negatively linked to MDA level. Consistently, Ref. [24] showed that medium energy (10.49 MJ/kg, M) during periparturient period in donkeys significantly enhanced average daily gain (ADG), tumor necrosis factor-α (TNF-α), CAT, total superoxide dismutase (T-SOD), glutathione peroxidase (GSH-Px), and total antioxidant capacity (T-AOC) levels in serum while reducing MDA, interleukin 1 (IL-1), IL-2, and IL-6 [24]. Furthermore, they revealed that beneficial rectal microbiota including *Candidatus_Saccharimonas*, *Fibrobacter*, *Lactobacillus* species, *Bifidobacterium* strains and *Akkermansia muciniphila* were positively correlated with antioxidant enzymes (CAT, GSH-Px, T-AOC, T-SOD) and negatively linked to inflammatory markers (IL-1, IL-2, IL-6, TNF-α). At the same time, higher energy level supplementation regulated bacteria like *norank_f_norank_o_Mollicutes_RF39*, *and norank_f_norank_o_Coriobacteriales* which are positively associated with inflammatory markers (IL-1, IL-2, IL-6, TNF-α), suggesting this bacterial group may promote inflammatory responses and excessive TNF-α production can lead to chronic inflammation and oxidative stress [24]. Consistently, a study examined how dietary energy levels affect meat donkey growth, focusing on cecal microbiota changes [109].

Low-energy diets reduced growth performance and nutrient digestibility while increasing oxidative stress. Cecal microbiome analysis revealed decreased *Firmicutes* and *Actinobacteria* but increased *Bacteroidetes* in low-energy groups.

Metabolomic changes involved energy metabolism pathways, suggesting microbiota-metabolism interactions influence growth outcomes [124]. Elevated oxidative stress and inflammation are key factors compromising immune response of animals during periparturient period [108,122]. An 82-day feeding trial utilizing fiber-to-concentrate ratios in total mixed rations significantly increased the abundance of beneficial intestinal bacteria, specifically *Firmicutes*, *Prevotella*, *Bacteroides*, *Proteobacteria*, and *Fibrobacter* [122]. The enrichment of these bacterial taxa was associated with upregulated galactose metabolism and glycolysis, suggesting enhanced host growth and metabolic function. Multienzyme (glyanase, β-mannanase, β-glucanase, cellulase, protease, and amylase) supplementation addition to basal diet in 2-month-old suckling donkeys significantly improved gut beneficial bacterial populations (*Firmicutes*, *Oscillospiraceae*, *Lachnospiraceae*, *Christensenellaceae*, *Christensenellaceae_R-7_group*) including Streptococcus in feces while reducing harmful Proteobacteria [99].

They found that though growth performance and digestibility showed minimal changes, the enhanced microbial balance provides crucial foundation for long-term digestive health during the critical weaning transition period [99]. Consistently, a study found Mannan oligosaccharides supplementation in 0.5 g/kg diet for 60 days significantly improved anti-inflammatory (decreased TNF-α, IL-6, and IL-17 levels) and antioxidant responses as well as metabolic health and beneficial microbiota (*Clostridium*, *Bacteroides*, *Parabacteroides*, *Lachnospiraceae_UCG-009* and *Faecalicoccus*) in Dezhou donkeys [123]. A study tested various concentrate feeding sequences in weaned donkeys and found significantly altered fecal microbiota composition and performance parameters [96].

The total mixed ration (TMR) group demonstrated superior growth performance, enhanced nutrient digestibility, and increased Firmicutes abundance compared to sequential feeding approaches. Firmicutes and Bacteroidetes dominated across all groups, with significant differences at genus level including *Treponema*, *Rikenellaceae-RC9-gut-group*, *Unidentified-F082*, and *Bacteroidales-RF16-group*. Volatile fatty acid profiles varied significantly, while predicted microbiota functions remained largely unchanged [101]. Consistently, ref. [25] documented a significantly positive effect of Yeast polysaccharide supplementation increasing the levels of immunoglobulin A (IgA) and immunoglobulin G (IgG) in Dezhou donkeys. In addition, Yeast polysaccharide positively promoted the fecal microbiome (enhanced levels of *Lactobacillus*, *Prevotella*, *Terriporobacter and Cellulosilyticum*) which were associated positively with metabolism and growth performance [25]. For ease of reference, the research development on microbiota association with production performance and health has been provided in Table 3. However, the microorganisms detected in feces can only represent the microbiota in the proximal rectum and cannot indicate the specific composition of the microbiota in the cecum and colon. In addition, though these are based primarily on fecal microbiota analysis which may not fully represent mucosa-associated communities where many host-microbe interactions occur. In future studies, technical approaches such as mucosal biopsies, comparative analysis of luminal microbiota and mucosal microbiota, and multi-omics integration can be used for further investigation.

## 7. Novel Insights and Future Directions for Donkey Nutrition Science

This review provides the first comprehensive mechanistic framework integrating donkey digestive physiology, gut microbiota ecology, and nutritional interventions to establish evidence-based feeding strategies. Unlike previous reviews that simply describe donkey nutrition or catalog microbiota compositions, this synthesis reveals causal pathways from dietary modifications through microbiota alterations to measurable health and production outcomes, representing a paradigm shift from empirical feeding practices to precision, microbiota-informed nutrition management.

### 7.1. Novel Contributions of This Review

This review has for the first time established a comprehensive framework that combines the nutritional requirements, digestive physiology and intestinal microbiota ecology of donkeys, systematically clarifying the mechanisms and pathways for optimizing the health and production performance of donkeys by regulating the intestinal flora through targeted nutritional intervention. The unique digestive physiological structure of donkeys, including high fiber digestibility and an energy supply mode centered on hindgut fermentation, determines the uniqueness of their nutritional management and it is not advisable to directly apply the feeding standards of horses or ruminants.

This article, by integrating multiple intervention studies, identified key microbial groups (such as *Akkermansia muciniphila*, *Prevotella*, *Ruminococcus*) that are continuously associated with health improvement, and revealed that nutritional interventions such as protein, methionine, and energy regulate the microbiota This further affects the potential pathways of donkey growth performance, antioxidant stress and immune inflammatory response. Meanwhile, the research clarified the non-linear dose-effect and key action Windows of nutritional intervention (such as the perinatal period and weaning period), providing a basis for the implementation of precision nutrition.

In addition, the review demonstrates that the utilization of unconventional feed resources (such as reed silage, bamboo leaves, and garlic by-products) based on local conditions can selectively regulate the microbiota through their bioactive components, thereby enhancing the economy and sustainability of the feeding system while ensuring production performance. It provides a theoretical basis and practical path for establishing an evidence-based and sustainable donkey breeding system.

Finally, we translate mechanistic knowledge into actionable management strategies through development of a microbiota-based nutritional optimization framework. This framework guides feed ingredient selection, energy level management, and fiber diversification based on predicted microbiota effects rather than crude nutrient composition alone, representing a fundamental departure from conventional donkey feeding practices. The regional adaptation principles and practical implementation solutions provided enable translation of research findings into field applications across diverse production contexts.

### 7.2. Critical Knowledge Gaps and Research Priorities

Despite significant advances, substantial knowledge gaps constrain optimization of donkey nutrition and limit precision of feeding recommendations. Species-specific nutritional requirements lack precise quantification. Current recommendations largely extrapolate from horse standards adjusted by body weight [83,91,92], ignoring fundamental differences in digestive physiology and metabolic efficiency. Priority research needs include systematic determination of maintenance energy requirements, protein and amino acid requirements across life stages, and macro/micromineral requirements specifically for donkeys under diverse environmental and production conditions. Such studies require controlled feeding trials with multiple treatment levels and precise measurement of intake, growth, milk production, and health outcomes, representing foundational work that remains incomplete for donkeys despite being well-established for horses, cattle, and other livestock species.

Beyond establishing nutritional requirements, mechanisms linking microbiota composition to specific health outcomes remain incompletely understood. While we establish associations between certain bacterial taxa and improved health markers [24,106,108], the precise metabolites, signaling molecules, and host-microbe interaction pathways mediating these effects require elucidation through mechanistic studies. Priority research includes metabolomic profiling linking microbial populations to specific metabolite profiles and host responses, metatranscriptomic analyses revealing functional activities of microbiota beyond taxonomic composition, controlled colonization studies testing causality of specific bacterial strains’ effects on donkey health, and host transcriptomic responses to microbiota manipulation to identify key host pathways influenced by gut microbes. Such multi-omics approaches would transform correlative observations into mechanistic understanding suitable for targeted interventions.

Compounding these challenges, individual variation in microbiota and nutritional responses limits precision of feeding recommendations. Geographic variation [98], breed differences, age-related changes [17,19], and individual animal variation in microbiota composition suggest that uniform feeding strategies may be suboptimal. Priority research should investigate genetic determinants of microbiota composition and feed efficiency to enable selective breeding for optimal microbiota, personalized nutrition approaches identifying individual animals’ specific nutritional needs based on microbiota profiling, factors determining resilience and stability of beneficial microbiota to enable selection of animals with robust microbiomes, and host-microbiome interactions determining response to dietary interventions to predict which animals will benefit most from specific feeding strategies.

Furthermore, long-term effects of nutritional interventions on microbiota stability and animal health remain unknown. Most studies examine short-term effects (60–82 days) [24,99,106,113,114], leaving questions about persistence of microbiota changes, potential adaptation or tolerance to interventions, and lifetime cumulative effects on health and longevity. Priority research includes longitudinal studies tracking microbiota and health outcomes across multiple years, multigenerational studies examining whether beneficial microbiota changes can be transmitted to offspring, and investigations of early-life nutritional programming effects on adult microbiota and health. Understanding temporal dynamics beyond immediate responses is essential for developing sustainable, long-term feeding strategies.

Another critical limitation involves practical, cost-effective methods for on-farm microbiota assessment and monitoring. Current microbiota profiling requires laboratory analysis impractical for routine production use. Priority research should develop and validate simplified fecal biomarkers (pH, specific VFA ratios, fecal score systems) that correlate with beneficial microbiota states, rapid, field-deployable diagnostic tools for assessing microbiota health status, decision support systems integrating available farm data (feed composition, performance metrics, simple fecal assessments) to predict microbiota status and recommend feeding adjustments, and economically optimized monitoring frequencies and sampling strategies balancing information value against cost. Such tools would enable practical implementation of microbiota-based nutrition management beyond research settings.

Finally, interactions between nutrition, microbiota, and management factors (housing, stress, disease, climate) require systematic investigation. Microbiota responses to nutritional interventions likely depend on concurrent environmental and management conditions, yet most feeding studies control or minimize such variation. Priority research should examine nutrition-stress interactions determining microbiota resilience to heat stress, cold stress, transport stress, and social stress, nutrition-disease interactions showing how microbiota modulation through feeding affects susceptibility to and recovery from infectious diseases, nutrition-environment interactions revealing how housing systems, seasonal variation, and geographic location influence optimal feeding strategies, and multi-factor optimization identifying feeding strategies that maintain microbiota health across diverse, real-world production conditions. Understanding these interactions is essential for robust, broadly applicable feeding recommendations.

### 7.3. Translational Impact and Future Vision

The framework established in this review enables a paradigm shift in donkey nutrition from empirical practices based on historical precedent and extrapolation from horses toward evidence-based, precision nutrition guided by understanding of digestive physiology and microbiota ecology. This transition has profound implications for sustainable donkey production systems worldwide. For donkey producers, particularly in developing regions where donkeys provide essential agricultural labor and income, microbiota-informed nutrition offers pathways to improve animal health, productivity, and welfare using regionally available feed resources. The demonstration that non-conventional feeds can maintain or enhance outcomes through beneficial microbiota modulation [40,41,46] reduces dependence on expensive purchased feeds, improving economic viability of donkey husbandry. The 15–25% improvements in growth performance and feed efficiency achieved through nutritional optimization [24,99,106,113] translate directly to reduced feeding costs and faster growth rates, significantly impacting producer profitability.

For donkey milk production, an emerging industry with growing recognition of donkey milk’s unique nutritional and therapeutic properties for human consumption [7,8,9], the evidence that lactation performance can be enhanced through microbiota modulation [25,114] provides scientific foundation for improving milk yield and quality. Understanding how maternal nutrition shapes offspring microbiota and health [123] enables optimization of breeding animal management to produce healthy foals with robust immune systems. For sustainable agriculture development, the successful integration of agricultural and processing waste materials (crop residues, garlic byproducts) into nutritious donkey feeds through microbiota-informed processing [33,38,41,61] demonstrates circular economy approaches reducing environmental impact while improving animal nutrition. This waste-to-feed valorization addresses both waste management and feed cost challenges simultaneously.

For animal welfare, preliminary studies have reported reductions in oxidative stress (20–40%) [111,124] and inflammatory responses (30–50%) [24] associated with nutritional optimization, though these findings, typically from studies with small sample sizes (*n* < 10) and short durations (30–60 days), require validation through larger-scale, longer-term research to establish their practical significance for animal health and comfort. The mechanistic understanding enables proactive nutritional management preventing health challenges rather than reactive treatment of nutritional diseases, representing a fundamental improvement in welfare standards. For veterinary medicine and animal health, recognition that many health challenges in donkeys (poor growth, low immunity, reproductive problems, metabolic stress) have nutritional-microbial etiologies creates opportunities for nutritional interventions as alternatives or complements to pharmaceutical treatments. The anti-inflammatory effects of optimal nutrition through microbiota modulation [24] suggest potential for nutritional management of inflammatory conditions, while the immune-enhancing effects [25,99,124] may reduce disease susceptibility and antibiotic usage.

Looking forward, the integration of emerging technologies (metagenomics, metabolomics, machine learning for predictive modeling, precision feeding systems) with the mechanistic frameworks established here promises continued advancement toward truly personalized nutrition for individual donkeys based on their specific microbiota profiles, genetic backgrounds, and production goals. Development of microbiota-targeted feed additives (precision prebiotics, specifically selected probiotics, postbiotics containing beneficial bacterial metabolites) could enable fine-tuned microbiota manipulation for specific health and production outcomes. However, realizing this vision requires sustained research investment addressing the critical knowledge gaps identified above, particularly in establishing species-specific nutritional requirements and elucidating causal mechanisms linking microbiota to health outcomes. It also requires commitment to translational research ensuring scientific advances reach end users through effective extension and education programs, accessible decision support tools, and practical feeding guidelines adapted to diverse production contexts.

## 8. Conclusions

This comprehensive review establishes that donkey nutrition must be understood not simply as providing nutrients to a passive recipient, but rather as orchestrating complex interactions between diet, gut microbiota, and host physiology to optimize health and productivity. The unique digestive adaptations that enable donkeys to thrive on low-quality feeds in harsh environments create both opportunities and requirements for specialized nutritional management distinct from horses and other livestock. By revealing mechanistic pathways from nutritional modifications through microbiota alterations to measurable outcomes, integrating findings across multiple studies to establish universal principles, and translating mechanistic knowledge into practical management strategies, this review provides the comprehensive framework needed to advance donkey nutrition from an understudied afterthought to a sophisticated, evidence-based discipline supporting sustainable production systems worldwide. The potential economic, social, and welfare impacts suggested by preliminary research through improved nutrition (indicated by short-term studies reporting 15–25% growth improvements, 20–40% oxidative stress reductions, and 30–50% inflammatory response decreases, though these findings require validation through larger-scale, longer-term studies) justify urgent prioritization of the research agenda outlined above to enable full realization of donkeys’ potential as efficient, resilient, and valuable livestock species. Currently, research on donkey nutrition is not a mainstream focus. Due to the small stock size of donkeys and their unique physiological and digestive characteristics, donkeys are often overlooked in the field of nutrition. However, the economic, edible, and medicinal values of donkeys have been increasing year by year alongside growing attention to the donkey industry. Unlike cattle, sheep, and horses (for which mature and comprehensive research systems have been developed), donkeys lack such a framework. Again, their small stock size and unique physiological digestion traits lead to their frequent neglect in nutrition studies. At present, the development of unconventional roughage specifically for donkeys is insufficient, and if existing nutritional research targeting ruminants is directly applied to donkeys, it will slow down or even hinder the development of the donkey industry. Regarding the future development of donkey nutrition research, the authors suggest that priority should be given to two key areas: first, the development and utilization of geographically sourced unconventional roughage for donkeys, and second, the systematic exploration of how donkey gut microbiota influences donkeys throughout the entire feeding period.

## Figures and Tables

**Table 1:** Conventional vs. non-conventional roughage options.

Feed Type	Category	Crude Fiber (%)	Crude Protein (%)	Advantages	Considerations	References
Garlic Byproducts	Non-conventional	Variable	Variable	Bioactive compounds, antimicrobial	Limited usage data	[42]
Alfalfa Hay	Conventional	25–30	10–19	High quality, good protein	More expensive	[61]
Corn Stalks	Conventional	>30	2–4	Readily available, low cost	Requires processing	[62]
Wheat Straw	Conventional	25–50	3–5	Common in northern regions	Low protein	[63]
Reed Silage	Non-conventional	Variable	Variable	Locally abundant, cost-effective	Requires fermentation	[62]
Bamboo Leaves	Non-conventional	High	Higher than straw	Antioxidant properties, drought-resistant	Limited research	[62]

**Table 3:** Nutritional interventions effects on donkey microbiota.

Treatment	Effects	Reference
Medium energy level (10.49 MJ/kg) during periparturient period	Enhanced average daily gain (ADG), TNF-α, CAT, T-SOD, GSH-Px, T-AOC levels Reduced MDA, IL-1, IL-2, IL-6 Beneficial rectal microbiota: *Candidatus_Saccharimonas*, *Fibrobacter*, *Lactobacillus*, *Bifidobacterium*, *Akkermansia muciniphila* These bacteria were positively associated with antioxidant enzymes and negative with inflammatory markers	[24]
Yeast polysaccharide supplementation	Increased immunoglobulin A (IgA) and immunoglobulin G (IgG) levels Enhanced fecal microbiome: *Lactobacillus*, *Prevotella*, *Terriporobacter*, *Cellulosilyticum* Positive association with metabolism and growth performance	[25]
Multienzyme supplementation (glyanase, β-mannanase, β-glucanase, cellulase, protease, amylase) in 2-month-old suckling donkeys	Improved beneficial bacterial populations: *Firmicutes*, *Oscillospiraceae*, *Lachnospiraceae*, *Christensenellaceae*, *Christensenellaceae_R-7_group*, *Streptococcus* Reduced harmful *Proteobacteria* Enhanced microbial balance for long-term digestive health during weaning	[101]
Total Mixed Ration (TMR) vs. sequential feeding in weaned donkeys	Superior growth performance and nutrient digestibility Increased *Firmicutes*, *Bacteroidetes*, *Treponema*, *Rikenellaceae-RC9-gut-group*, *Unidentified-F082*, *Bacteroidales-RF16-group* *Varied volatile fatty acid profiles (Acetic Acid*, *Propionic Acid*, *Butyric Acid*, *Isobutyric Acid*, *Valeric Acid*, *Isovaleric Acid)*	[103]
Concentrate supplements with soybean meal (donkey foals)	Enhanced beneficial gut microbiota: *Akkermansia*, *Oscillospiraceae*, *Porphyromonas*, *Streptococcus* Improved growth performance, serum hormones, and metabolites Superior effects on gut health	[106]
Dietary protein supplementation (12.52%)	Modulated hindgut microbiota including *Prevotella*, *Clostridiumsensustricto1*, NK4A214 group, OscillospiraceaeUCG-002, OscillospiraceaeUCG-005 Enhanced microbial community composition Improved nutrient digestibility and overall performance	[107]
Methionine supplementation	5 g/d: Increased abundance of *Methanocorpusculum* and *Ruminococcus* 15 g/d: Altered gut microbiota including *Ruminococcus*, *Peptococcus*, *Anaeroplasma* Reduced oxidative stress (lower MDA) Enhanced antioxidant capacity (higher T-AOC and CAT activity) Beneficial bacteria positively associated with antioxidant activity	[108]
Low-energy diets	Reduced growth performance and nutrient digestibility Increased oxidative stress Cecal microbiome changes: decreased *Firmicutes* and *Actinobacteria*, increased *Bacteroidetes* Metabolomic changes in energy metabolism pathways	[109]
Fiber-to-concentrate ratios in total mixed rations (82-day trial)	Increased abundance of beneficial intestinal bacteria: *Firmicutes*, *Prevotella*, *Bacteroides*, *Proteobacteria*, *Fibrobacter* Upregulated galactose metabolism and glycolysis Enhanced host growth and metabolic function	[110]
Mannan oligosaccharides (0.5 g/kg diet for 60 days)	Improved anti-inflammatory response: decreased TNF-α, IL-6, IL-17 levels Enhanced antioxidant responses and metabolic health Increased beneficial microbiota: *Clostridium*, *Bacteroides*, *Parabacteroides*, *Lachnospiraceae_UCG-009*, *Faecalicoccus*	[111]
Dietary energy level (Low and high energy feed supplementation)	Both the diet significantly enhanced growth performance including ADG and feed efficiency Enhanced beneficial bacteria such as *Firmicutes*, *Bacteroidetes*, *unidentified_Prevotellaceae*, *unidentified_Ruminococcaceae* with improved metabolism and growth performance of donkeys ameliorated growth performance of donkeys. The effect of High energy on all parameters including microbiota and growth performance was more significant compared to low energy levels.	[112]

## Data Availability

No new data were created or analyzed in this study.
